# Chemistry, Physiology and Pathology of Nitrous Oxide—Practical Points

**Published:** 1889-03

**Authors:** 


ARTICLE V.

### CHEMISTRY, PHYSIOLOGY AND PATHOLOGY OF NITROUS OXIDE—PRACTICAL POINTS.

---

### GASEOUS INTERCHANGE.

And, first, as to the effects produced by the inhalation upon the gases contained in the lungs and blood, and for which I have used the term "gaseous interchange."

1. That the expired gas tends to become precisely similar in composition to that inspired, so that, *prima facie*, one would be justified in concluding that no very active decomposition of the gas occurs; and this conclusion is further justified by the researches of Hermann and other observers.

2. That the carbonic acid, instead of being increased in quantity, as is the case in ærial expirations, is rapidly and continuously diminished.

3. That the amount of oxygen is diminished and, in fact, almost entirely disappears.

4. That, as might be expected from the fact, the nitrogen does not take any very active part in the physiological functions, even under ordinary circumstances, in gaseous inhalation, it is removed by diffusion alone, and hence very slowly.

5. That the substitution of nitrous oxide for the other gases does not take place at all equally or proportionately, but mainly at the expense of the oxygen, which is rapidly reduced.

Dr. Amory's experiments upon himself and upon dogs, have very much the same result, but were carried somewhat further. He found that the relative amount of carbonic acid inhaled during fifty respirations of the gas, was only about half of that found in the same number of ærial respirations.

### CONCLUSIONS.

1. That the progressive loss of carbonic acid in the expired gas is not associated with any accumulation in the blood, and must, therefore, be due either to lessened production in the tissues or to defective absorption by the blood.

2. Now, in order that carbonic acid may be produced, free oxygen, or an agent capable of yielding free oxygen, must be present. But we see that the free oxygen is displaced by nitrous oxide, and we know that the latter undergoes no decomposition in the blood.

3. Hence, during Inhalation, the process of tissue metabolism and the production of free carbonic acid is in abeyance.

### PULSE TRACINGS.

A comparison of the pulse trace taken from an average healthy individual during the height of anæsthesia, with one taken from the same individual immediately prior to inhalation, shows:

1. An actual increase in the number of heart beats.
2. The greater height of the stroke.
3. The greater sharpness of the initial curve and apices.
4. The almost complete absence of the tidal wave.
5. The accentuation of the dicrotic wave, which is at the same time removed further from the apex of the trace.

I think we may gather from the consideration of these tracings alone, (1) that the heart's action is accelerated and slightly increased in force; (2) that the blood-pressure is lowered; and this latter point has been proved by actual experiments.

### CAUSES OF DEATH.

1. That when death has occurred it has been due either to syncope or asphyxia.

2. Simple faintness may occur either before the inhalation is complete, apparently from fright, or during the stage of recovery, apparently from the shock of the operation.

3. Fatal syncope has, with very few exceptions, been distinctly traceable to the shock, consequent upon commencing or continuing the operation when the anæsthesia was incomplete, and have, as may readily be imagined, occurred in patients more or less out of health.

4. Hence the importance of never permitting any operation in a semi-anæsthetical condition.

5. The occurrence of asphyxia from laryngeal spasm, or from falling together of the epiglottidean folds, as in chloroform, has not been recorded with regard to nitrous oxide, and does not seem likely to give rise to any trouble. Such a condition, if it does occur, is, of course, to be looked for during the height of anæsthesia.

6. Fatal asphyxia has invariably been the result of some mechanical cause, *e. g.*, regurgitation of vomited matter, slipping of tooth or gag into the larynx, and has therefore usually occurred during or immediately after the completion of the operation.

7. Hence the importance of watching the mouth, as well as the face, during the stage of recovery.

SYNCOPE.

*Recognized by*—Sudden dilatation of pupil, extreme pallor, heart failure, muscular relaxation, feebleness of breathing.

*Treatment.*—Prone position, draw out the tongue; artificial respiration.

ASPHYXIA.

*Recognized by*—Increasing duskiness, violent efforts at respiration, gradual failure of pulse.

*Treatment.*—Remove foreign bodies (inversion), or draw the tongue *well* forward, press on the chest, wipe out mucous; laryngotomy followed by artificial respiration.—SILK, *Dental Record.*

